# Changes in salivary biomarkers of pain, anxiety, stress, and inflammation related to tooth movement during orthodontic treatment: a systematic review

**DOI:** 10.1590/2177-6709.29.6.e242436.oar

**Published:** 2024-12-16

**Authors:** Rodrigo RODRIGUES, Caio Melo MESQUITA, Helena Benatt do Nascimento ALVES, Filipe Gontijo SILVA, Walbert de Andrade VIEIRA, Paula Cristiana Santos de AGUIAR, Carlos FLORES-MIR, Luiz Renato PARANHOS, Rui Barbosa de BRITO-JÚNIOR

**Affiliations:** 1 São Leopoldo Mandic College, Dentistry Course (Campinas/SP, Brazil).; 2 Federal University of Uberlândia, Dentistry Course (Uberlândia/MG, Brazil).; 3 State University of Campinas, Dentistry Course, Endodontics Department (Campinas/SP, Brazil).; 4 University of Alberta, School of Medicine and Dentistry, Department of Dentistry (Edmonton, Canada).; 5 Federal University of Uberlândia, Dentistry Course, Department of Preventive and Social Dentistry (Uberlândia/MG, Brazil).; 6 São Leopoldo Mandic School of Dentistry, Department of Molecular Biology (Campinas/SP, Brazil).

**Keywords:** Orthodontics, Saliva, Biomarkers, Ortodontia, Saliva, Biomarcadores

## Abstract

**Objective:**

This systematic review aimed to analyze the literature on changes in endogenous salivary biomarkers of pain, anxiety, stress, and inflammation related to tooth movement during orthodontic treatment of children and adolescents.

**Material and Methods:**

An electronic search was performed in nine databases to identify quasi-experimental studies, without restricting publication language and year. Two reviewers extracted the data and assessed the individual risk of bias using the JBI tools, and the certainty of evidence using the GRADE tool.

**Results:**

The electronic search found 7,038 records, of which 12 met the eligibility criteria and were included in the qualitative synthesis. Most studies had a low risk of bias. Biomarkers were grouped into five categories: electrolytes, enzymes, hormones, immunoglobulins, and mediators. Electrolytes showed decreased Ca2+, Pi3+ and K+ levels, and increased Na+ and Cl- levels. All enzymes (ALP, LDH, MMP8, and MMP9) increased over time. Hormones presented a decrease in leptin and some fluctuations in daily cortisol levels. Immunoglobulins (IgA, IgG, IgM, IgD, and IgE) had no significant changes, and salivary IgA showed divergent results among studies. Mediators (sRANKL, OPG, IL‐1β, and PGE2) showed fluctuations at different treatment stages, mainly after orthodontic activation.

**Conclusions:**

Based on a very low certainty level, orthodontic tooth movement had little to no effect on endogenous salivary biomarkers.

## RESUMO

**Objetivo:**

Esta revisão sistemática teve como objetivo analisar a literatura sobre alterações em biomarcadores salivares endógenos de dor, ansiedade, estresse e inflamação relacionados à movimentação dentária durante tratamento ortodôntico de crianças e adolescentes.

**Material e Métodos:**

Foram realizadas buscas eletrônicas em nove bases de dados para identificar estudos quase-experimentais sobre o tema, sem restrições de idioma e ano de publicação. Dois revisores extraíram os dados e avaliaram o risco individual de viés usando as ferramentas JBI, e a certeza da evidência usando a ferramenta GRADE.

**Resultados:**

A busca eletrônica encontrou 7.038 registros, dos quais 12 atenderam aos critérios de elegibilidade e foram incluídos na síntese qualitativa. A maioria dos estudos apresentou baixo risco de viés. Os biomarcadores foram agrupados em cinco categorias: eletrólitos, enzimas, hormônios, imunoglobulinas e mediadores. Os eletrólitos apresentaram diminuição dos níveis de Ca2+, Pi3+ e K+ e aumento dos níveis de Na+ e Cl-. Todas as enzimas (ALP, LDH, MMP8 e MMP9) aumentaram ao longo do tempo. Os hormônios apresentaram diminuição da leptina e algumas oscilações nos níveis diários de cortisol. As imunoglobulinas (IgA, IgG, IgM, IgD e IgE) não tiveram alterações significativas, e a IgA salivar apresentou resultados divergentes entre os estudos. Os mediadores (sRANKL, OPG, IL-1β e PGE2) apresentaram flutuações nas diferentes fases do tratamento, principalmente após a ativação ortodôntica.

**Conclusões:**

Com base em um nível de certeza muito baixo, a movimentação dentária ortodôntica teve pouco ou nenhum efeito sobre biomarcadores salivares endógenos.

## INTRODUCTION

The proteomic study of salivary samples is relevant for detecting orthodontic treatment biomarkers and for revealing possible new pathogenetic mechanisms in young and adult patients.^1^

Regarding orthodontic and orthognathic treatment, salivary proteins can indicate the effectiveness and adverse consequences of orthodontic treatment, such as treatment-induced external root resorption.^2^ Among eight proteins studied with altered expression during orthodontic tooth movement, four (S100-A9 protein, Ig-J chain, region C of Ig-α1 chain, and CRISP3) have known roles in inflammation and bone resorption, and together they potentially monitor the progression of orthodontic treatment.^3^

Conversely, analyzing pain-related biomarkers at isolated moments has limitations in measuring temporary pain experiences usually associated with chewing limitations in orthodontic patients.^4^ However, in clinically healthy individuals, orthodontic treatment modifies the salivary oxidative-antioxidative balance, and the increased concentration of nickel in saliva released by orthodontic appliances is associated with these changes.^5^ Although orthodontic tooth movement and materials alter some gingival crevicular fluid and saliva biomarkers, the differences do not exceed physiological limits or appear to suggest oxidative damage.^6^

Patients undergoing orthodontic treatment also show a significant increase in bleeding on probing, MMP8, and MMP9 one week after orthodontic appliance placement, and a decrease one month after periodontal treatment. These parameters evaluate the periodontal status of orthodontic patients.^7^ Salivary MMP9 accurately predicts the level of periodontal inflammation during orthodontic treatment, which is also associated with malocclusion type.^8^ Combining periodontal and orthodontic treatments decreased salivary IL-1β values significantly, compared to isolated periodontal therapy, with a perceived reduction in all treated Angle classes and higher benefits for Angle Class III.^9^

The stress and anxiety responses include hypothalamus-pituitary-adrenal (HPA) axis activation, causing cortisol release.^10^ Salivary cortisol reflects the amount of cortisol that escapes such binding proteins and enters the salivary glands and saliva, also called bioavailable.^11^ Tooth movement discomfort during orthodontic treatment may trigger anxiety and stress.^12^ A previous study demonstrated statistically significant differences in state-trait anxiety levels between pre- and post-rapid maxillary expansion stages.^13^

Children and adolescents undergoing orthodontic treatment have clinical experiences related to orthodontic movement, such as the perception of pain, anxiety, stress, and inflammation of periodontal tissues. The analysis and consolidation of information on biomolecular changes possibly associated with these clinical phenomena may support the guidance and management of cases and research development to improve orthodontic treatment. However, current evidence is uncertain on the magnitude of changes in salivary biomarker levels or whether these changes occurred during the whole orthodontic treatment.

Therefore, the present review aimed to systematically analyze the literature on changes in salivary biomarkers of pain, anxiety, stress, and inflammation related to tooth movement during orthodontic treatment of children and adolescents.

## MATERIAL AND METHODS

### PROTOCOL AND REGISTRATION

The protocol was reported according to the Preferred Reporting Items for Systematic Review and Meta-Analysis Protocols (PRISMA-P),^14^ and registered in the International Prospective Register of Systematic Reviews (PROSPERO) database under number CRD42023428830 (https://www.crd.york.ac.uk/PROSPERO/). This systematic review was reported following the Preferred Reporting Items for Systematic Reviews and Meta-Analyses (PRISMA),^15^ and conducted according to the Joanna Briggs Institute (JBI) Manual.^16^

### RESEARCH QUESTION AND ELIGIBILITY CRITERIA

This review was designed to answer the following question: *“Is tooth movement during orthodontic treatment (intervention) related to changes in endogenous salivary biomarkers of pain, anxiety, stress, and inflammation (outcome) in children and adolescents (population)?”*. It followed the PICO framework: P (population), I (intervention), C (comparison), and O (outcome).

#### Inclusion criteria

a) Population: Mixed and permanent dentition patients, including children and adolescents up to 19 years old, according to the WHO.
b) Intervention: Any orthodontic treatment related to tooth movement produced by fixed or removable orthodontic appliances.
c) Comparison: Children and adolescents who have not undergone orthodontic treatment (comparator group) / Children and adolescents before undergoing orthodontic treatment.
d) Outcome: Changes in endogenous salivary biomarkers of pain, anxiety, stress, and inflammation, such as electrolytes, enzymes, hormones, immunoglobulins, and mediators.
e) Study design: Quasi-experimental studies (e.g., studies with the same participants evaluated before and after the intervention). That included cross-sectional, longitudinal, and cross-sequential studies with at least one pre-treatment measurement, and no restrictions regarding laboratory data processing and analysis methods.
f) There were no restrictions on publication language or year.

#### Exclusion criteria

a) Studies without whole saliva sample collection and analysis (e.g., studies with only gingival crevicular fluid samples).
b) Studies with patients with diseases or conditions that represent potential confounding factors in salivary biomarker collection and/or analysis.
c) Studies with sample overlapping (in this case, considering the most recent study that best described the methodology and results).
d) Books, book chapters, case reports, case series, event papers, editorials, letters to the editor, literature reviews, qualitative studies, and animal studies.

## SOURCES OF INFORMATION, SEARCH, AND SELECTION OF STUDIES

The electronic searches were performed on April 2023 in Embase, LILACS (Latin American and Caribbean Health Science Literature), BBO (Brazilian Bibliography of Odontology), MedLine (via PubMed), and SciELO databases. The Scopus and Web of Science citation databases were also searched. The EASY and ProQuest databases partially captured the gray literature. These steps minimized the selection bias. The MedLine search was constantly updated with electronic alerts until September 2023.

The search descriptors were selected according to the MeSH (Medical Subject Headings), DeCS (Health Sciences Descriptors), and Emtree (Embase Subject Headings) resources. The Boolean operators “AND” and “OR” promoted several combinations among the descriptors, respecting the syntax rules of each database. Table 1 shows more details of search strategies and databases.

**Table 1:** Database search strategies.

Database	Search strategies (April 2023) and update (September 2023)
	Main databases
Embase https://www.embase.com	(‘child’/exp OR ‘child’ OR ‘children’/exp OR ‘children’ OR ‘adolescent’/exp OR ‘adolescent’ OR ‘adolescence’/exp OR ‘adolescence’ OR ‘teen’ OR ‘teenager’/exp OR ‘teenager’ OR ‘youth’/exp OR ‘youth’) AND (‘orthodontics’/exp OR ‘orthodontics’ OR ‘orthodontic treatment’/exp OR ‘orthodontic treatment’ OR ‘orthodontics, preventive’/exp OR ‘orthodontics, preventive’ OR ‘orthodontics, interceptive’/exp OR ‘orthodontics, interceptive’ OR ‘orthodontics, corrective’/exp OR ‘orthodontics, corrective’ OR ‘maxillary expansion’/exp OR ‘maxillary expansion’ OR ‘orthodontic appliances, fixed’/exp OR ‘orthodontic appliances, fixed’ OR ‘orthodontic appliances, functional’/exp OR ‘orthodontic appliances, functional’ OR ‘orthodontic appliances, removable’/exp OR ‘orthodontic appliances, removable’ OR ‘orthodontic appliances’/exp OR ‘orthodontic appliances’) AND (‘saliva’/exp OR ‘saliva’ OR ‘salivary changes’ OR ‘salivary composition’ OR ‘biomarkers’/exp OR ‘biomarkers’ OR ‘salivary biomarkers’ OR ‘pain’/exp OR ‘pain’ OR ‘anxiety’/exp OR ‘anxiety’ OR ‘stress’/exp OR ‘stress’ OR ‘inflammation’/exp OR ‘inflammation’ OR ‘inflammation mediators’/exp OR ‘inflammation mediators’ OR ‘cytokines’/exp OR ‘cytokines’) AND [embase]/lim
LILACS and BBO http://lilacs.bvsalud.org/	((“Child” OR “Children” OR “Adolescent” OR “Adolescence” OR “Teen” OR “Teenager” OR “Youth”) AND (“Orthodontics” OR “Orthodontic Treatment” OR “Orthodontics, Preventive” OR “Orthodontics, Interceptive” OR “Orthodontics, Corrective” OR “Maxillary Expansion” OR “Orthodontic Appliances, Fixed” OR “Orthodontic Appliances, Functional” OR “Orthodontic Appliances, Removable” OR “Orthodontic Appliances”) AND (“Saliva” OR “Salivary Changes” OR “Salivary Composition” OR “Biomarkers” OR “Salivary Biomarkers” OR “Pain” OR “Anxiety” OR “Stress” OR “Inflammation” OR “Inflammation Mediators” OR “Cytokines”)) AND ( db:(“LILACS” OR “BBO”))
MEDLINE (via PubMed) http://www.ncbi.nlm.nih.gov/pubmed	#1 “Child”[Mesh] OR “Children”[tw] OR “Adolescent”[Mesh] OR “Adolescence”[tw] OR “Teen”[tw] OR “Teenager”[tw] OR “Youth”[tw] #2 “Orthodontics”[Mesh] OR “Orthodontic Treatment”[tw] OR “Orthodontics, Preventive”[Mesh] OR “Orthodontics, Interceptive”[Mesh] OR “Orthodontics, Corrective”[Mesh] OR “Maxillary Expansion”[tw] OR “Orthodontic Appliances, Fixed”[Mesh] OR “Orthodontic Appliances, Functional”[Mesh] OR “Orthodontic Appliances, Removable”[Mesh] OR “Orthodontic Appliances”[Mesh] #3 “Saliva”[Mesh] OR “Salivary Changes”[tw] OR “Salivary Composition”[tw] OR “Biomarkers”[Mesh] OR “Salivary Biomarkers”[tw] OR “Pain”[Mesh] OR “Anxiety”[Mesh] OR “Stress”[tw] OR “Inflammation”[Mesh] OR “Inflammation Mediators”[Mesh] OR “Cytokines”[Mesh]
	#1 AND #2 AND #3
SciELO https://scielo.org/	#1 “Child” OR “Children” OR “Adolescent” OR “Adolescence” OR “Teen” OR “Teenager” OR “Youth” #2 “Orthodontics” OR “Orthodontic Treatment” OR “Orthodontics, Preventive” OR “Orthodontics, Interceptive” OR “Orthodontics, Corrective” OR “Maxillary Expansion” OR “Orthodontic Appliances, Fixed” OR “Orthodontic Appliances, Functional” OR “Orthodontic Appliances, Removable” OR “Orthodontic Appliances” #3 “Saliva” OR “Salivary Changes” OR “Salivary Composition” OR “Biomarkers” OR “Salivary Biomarkers” OR “Pain” OR “Anxiety” OR “Stress” OR “Inflammation” OR “Inflammation Mediators” OR “Cytokines”
	#1 AND #2 AND #3
Scopus http://www.scopus.com/	( TITLE-ABS-KEY ( “Child” OR “Children” OR “Adolescent” OR “Adolescence” OR “Teen” OR “Teenager” OR “Youth” ) AND TITLE-ABS-KEY ( “Orthodontics” OR “Orthodontic Treatment” OR “Orthodontics, Preventive” OR “Orthodontics, Interceptive” OR “Orthodontics, Corrective” OR “Maxillary Expansion” OR “Orthodontic Appliances, Fixed” OR “Orthodontic Appliances, Functional” OR “Orthodontic Appliances, Removable” OR “Orthodontic Appliances” ) AND TITLE-ABS-KEY ( “Saliva” OR “Salivary Changes” OR “Salivary Composition” OR “Biomarkers” OR “Salivary Biomarkers” OR “Pain” OR “Anxiety” OR “Stress” OR “Inflammation” OR “Inflammation Mediators” OR “Cytokines” ) )
Web of Science http://apps.webofknowledge.com/	#1 TS=(“Child” OR “Children” OR “Adolescent” OR “Adolescence” OR “Teen” OR “Teenager” OR “Youth”) #2 TS=(“Orthodontics” OR “Orthodontic Treatment” OR “Orthodontics, Preventive” OR “Orthodontics, Interceptive” OR “Orthodontics, Corrective” OR “Maxillary Expansion” OR “Orthodontic Appliances, Fixed” OR “Orthodontic Appliances, Functional” OR “Orthodontic Appliances, Removable” OR “Orthodontic Appliances”) #3 TS=(“Saliva” OR “Salivary Changes” OR “Salivary Composition” OR “Biomarkers” OR “Salivary Biomarkers” OR “Pain” OR “Anxiety” OR “Stress” OR “Inflammation” OR “Inflammation Mediators” OR “Cytokines”)
	#1 AND #2 AND #3
Gray literature	
EASY https://easy.dans.knaw.nl/	(“Orthodontics” OR “Orthodontic Treatment” OR “Orthodontic Appliances”) AND (“Saliva” OR “Salivary Changes” OR “Salivary Composition” OR “Biomarkers” OR “Cytokines”)
ProQuest https://www.proquest.com/	(“Orthodontics” OR “Orthodontic Treatment” OR “Orthodontic Appliances”) AND (“Saliva” OR “Salivary Changes” OR “Salivary Composition” OR “Biomarkers” OR “Cytokines”)

The results were exported to EndNote Web™ software (Clarivate™ Analytics, Philadelphia, USA), in which duplicates were removed automatically and the remaining ones, manually. The other results were exported to Rayyan QCRI (Qatar Computing Research Institute, Doha, Qatar),^17^ for the study selection phase. The manual analysis of the gray literature occurred simultaneously and fully using Microsoft Word™ 2010 (Microsoft™ Ltd., Washington, USA).

Two reviewers [R.R. and C.M.M.] performed a calibration exercise before selecting the studies. They discussed the eligibility criteria and applied them to a sample of 20% of the retrieved studies, to determine inter-examiner agreement. The selection started after reaching an adequate level of agreement (Kappa ≥ 0.81) and occurred in two phases.

In the first phase, two eligibility reviewers [R.R. and C.M.M.] methodically analyzed the titles and abstracts of the studies independently. A third examiner investigated and solved disagreements between the reviewers. Titles unrelated to the topic and abstracts were eliminated in this phase, respecting the eligibility criteria. In the second phase, the full texts of the preliminarily eligible studies were obtained and evaluated. 

If the full texts were not found, a bibliographic request was made to the library database (COMUT), and an e-mail was sent to the corresponding authors to obtain the texts.

### DATA COLLECTION

A calibration exercise was performed before data extraction, to ensure consistency between the reviewers, in which the data from three eligible studies were extracted jointly. After the calibration, two reviewers [R.R. and C.M.M.] independently and blindly extracted the data from the eligible studies. A third reviewer analyzed the conflicts in cases of disagreements in data extraction.

The following data were extracted from the articles: study characteristics (authors, publication year, title, publication journal, impact factor, country/region of study performance, study design, ethical criteria, application of consent and/or permission forms, funding, and conflicts of interest), sample characteristics (number of participants, sex, age, ethnicity, eligibility criteria, study groups, and type of orthodontic appliance and time of use), data collection and processing characteristics (salivary sample collection and analysis methods, salivary biomarkers, and statistical test), and main results (objectives, salivary biomarker changes, and primary outcomes). The impact factor was collected from the 2023 Incites Journal Citation Reports (JCR, Clarivate Analytics) metrics. In case of incomplete or insufficient data, the corresponding authors were contacted via e-mail, up to three times at weekly intervals.

### RISK OF BIAS ASSESSMENT

Two reviewers [W.A.V. and L.R.P.] independently assessed the risk of bias in the selected studies using the JBI Critical Appraisal Tools for use in JBI Systematic Reviews - Checklist for Quasi-Experimental Studies.^18^ Any disagreements were resolved by discussing and consulting with a third reviewer.

### SUMMARY MEASURES AND SYNTHESIS OF RESULTS

The data collected from the selected studies were organized in spreadsheets on Microsoft Excel™ 2019 (Microsoft™ Ltd., Washington, USA) and described narratively (qualitative synthesis). The quantitative results of salivary biomarker levels for each assessed time in the selected studies were collected and analyzed. A meta-analysis was planned but not performed, due to the high heterogeneity of studies.

### CERTAINTY OF EVIDENCE (GRADE APPROACH)

Two reviewers [W.A.V. and L.R.P.] independently ranked the overall strength of evidence, using the Grading of Recommendations, Assessment, Development, and Evaluation (GRADE) tool.^19^ The authors followed the adaptations by Murad et al.^20^ to assess the criteria in systematic reviews without meta-analyses.

## RESULTS

### STUDY SELECTION

The electronic search identified 7,038 results distributed across nine electronic databases, including the gray literature. After removing duplicates, 4,899 results remained for analysis. A careful reading of titles and abstracts excluded 4,864 results. Five records could not be retrieved for full-text reading.

**Figure 1:**
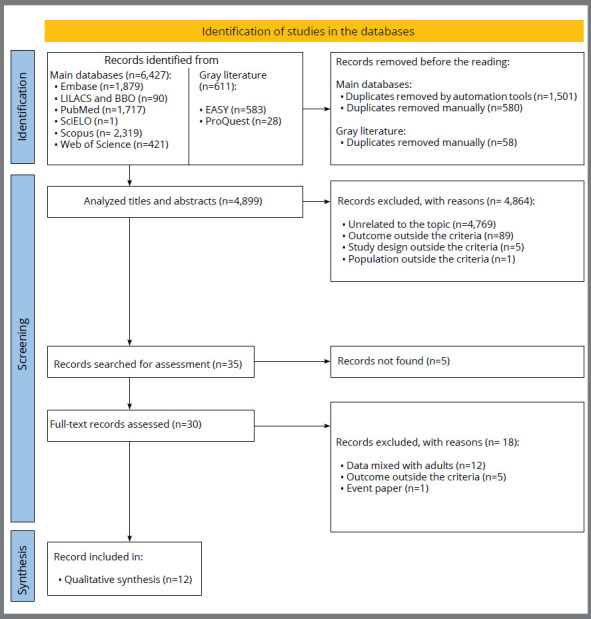
Flowchart of study selection.

After reading the full texts, 18 studies were excluded, and 12^13^ ^,^ ^21^ ^-^ ^31^ were included in the qualitative synthesis. Cohen’s Kappa coefficient obtained during study selection was 0.975, indicating an excellent level of inter-reviewer reliability. That corresponds to only 1.25% (61 of 4899) divergences between reviewers. Figure 1 details the study selection process.

### STUDY AND SAMPLE CHARACTERISTICS

The articles were published from 1984 to 2023 and performed in ten countries, with five studies in Asia,^22^ ^,^ ^25^ ^,^ ^26^ ^,^ ^28^ ^,^ ^29^ two in Europe,^30^ ^,^ ^31^ two in a transcontinental Asia-Europe country (Turkey)^13^ ^,^ ^27^, two in South America,^23^ ^,^ ^24^ and one in Africa.^21^ The sum of eligible study participants resulted in 249 patients. Age groups in eligible studies ranged from eight^29^ to 18 years old,^24^ ^,^ ^28^ ^,^ ^31^ and male patients comprised most studies sampling by sex. Only one study did not report the age range,^25^ and another did not test by sex.^29^ Three studies^23^ ^,^ ^24^ ^,^ ^28^ included groups of adults, but data collection and synthesis considered only the young group. 

Eleven studies^13^ ^,^ ^21^ ^-^ ^25^ ^,^ ^27^ ^-^ ^31^ used fixed orthodontic appliances, one used a rapid maxillary expansion appliance,^13^ and one used a removable appliance.^26^ Orthodontic treatment lasted from seven days^23^ to 32 months^21^ for study analyses.

The data collected on saliva collection methods included three main steps: study participant preparations and saliva sample collection and storage. Two studies lacked preparation data,^28^ ^,^ ^31^ and one did not report storage data.^13^ Each study applied different saliva analysis methods according to each analyzed biomarker. Seven studies^23^ ^-^ ^25^ ^,^ ^28^ ^-^ ^31^ used ELISA kits for each specific biomarker.

The analyzed biomarkers were salivary IgA in three studies;^21^ ^,^ ^23^ ^,^ ^29^ cortisol in two studies;^13^ ^,^ ^27^ immunoglobulins IgG, IgM, IgD, and IgE in one study;^21^ calcium (Ca2+), phosphate (Pi3+), sodium (Na+), chloride (Cl-), and potassium (K+) in one study;^22^ soluble receptor activator of nuclear factor Kappa B ligand (sRANKL) and osteoprotegerin (OPG) in one study;^24^ leptin in one study;^25^ alkaline phosphatase (ALP) and lactate dehydrogenase (LDH) in one study;^26^ interleukin-1β (IL-1β) and prostaglandin E2 (PGE2) in one study;^28^ bone morphogenetic protein 4 (BMP4) in one study;^30^ and matrix metalloproteinases 8 and 9 (MMP8, MMP9) in one study.^31^

Table 2 shows the main characteristics of each eligible study.

**Table 2:** Main characteristics of the eligible studies.

Author, year (Country) Journal of publication (Impact factor) [Analyzed biomarkers]	Study design # Ethical criteria - term $ Funding * Conflicts of interest	Sample size (n) Sex (♀, ♂) Age group (Mean age)	Group (n) | Group (n) Orthodontic appliance (Usage time)	Method of saliva collection - Preparation - Collection - Storage	Method of saliva analysis
Ganhão et al.^21^, 1984 (South Africa) J Dent Assoc S Afr (N.R.) [IgA; IgG; IgM; IgD; IgE]	Prospective QE # N.R. - N.R.; $ N.R.; * N.R.	(n = 31) (♀ = 22, ♂ = 9) 11-15 yo (N.R.)	Only group (n=31) Fixed orthodontic appliance. (12-32 months)	- Mouth rinses, relax 5min, swallow saliva, lower head, and wait 5min. - UWS collected into a cylinder with 1mg of sodium azide. - Mixed and stored airtight at -10°C until assayed.	- Simplified Radial Immunodiffusion Technique (for IgA, IgG, IgM e IgD); - Phadabas IgE RIA Test Kit.
Li et al.^22^, 2009 (China) J Oral Rehabil (2.9) [Ca2+; Pi3+; Na+; Cl-; K+]	Prospective QE # Yes - Consent term; $ National Natural Science Foundation of China (No. 30430690); * None.	(n = 24) (♀ = 12, ♂ = 12) 10-16 yo (N.R.)	G1 (n=12) | Comparator group (n=12) Fixed orthodontic appliance: OPA-K (Tomy Co., Tokyo, Japan) straight archwires for both arches. (6 months)	- Overnight fasting and collection in the morning between 9am and 11am. - UWS (5mL) spit into a cylinder for 10min. - Centrifugation for 5min at 690g at room temperature and the supernatant was analyzed.	- Automated Biochemistry Analyzer (7060 type; Hitachi Co., Tokyo, Japan).
Campos et al.^23^, 2010 (Brazil) Med Sci Monitor (3.1) [IgA]	Prospective QE # Yes - Consent term; $ N.R.; * N.R.	(n = 10) (♂ = 10) 11-13 yo (12.2±0.7)	Young group (n=10) Fixed orthodontic appliance: brackets and tubes (Mini Standard Edgewise; American Orthodontics, Sheboygan, WI, USA), 0.014-in straight archwire (Titanium Memory Wire, 857-504; American Orthodontics), and 0.010mm steel wire. (7-14 days)	- Collection on waking, before toothbrushing, drinking, or eating. - Swab under tongue for 2min and transferred to the Salivette Cortisol device (51.1534.500; Sarstedt, Numbrecht, Germany). - Domestic freezer up to 48h, cold boxes with ice for transportation and at -80°C up to analysis.	- Human IgA ELISA Quantitation Kit (Bethyl Laboratories, Montgomery, TX, USA); - Spectramax 190 Microtiter Plate Absorbance Reader (Molecular Device, Sunnyvale, CA, USA).
Gecgelen et al.^13^, 2012 (Turkey) J Oral Rehabil (2.9) [Cortisol]	Prospective QE # Yes - Consent term; $ Süleyman Demirel University Scientific Research Projects Unit (No. 1880- D-09); * N.R.	(n = 40) (♀ = 20, ♂ = 20) 10.9-14.7 yo (N.R.)	Only group (n=40) Peak (n=19) | Post-peak (n=21) Fixed orthodontic appliance: rapid maxillary expansion appliance with acrylic bonding. (36 days)	- No drinking (only water allowed) or eating for 1h, and sit in normal position. - Saliva collected into a polypropylene tube for 3min. - Storage: N.R.	- Electrochemi-luminescence immunoassay (ECLIA); - Cobas Cortisol Kit (Roche Diagnostics, Indianapolis, IN, USA); - Elecsys 2010 immunoanalyser system (Roche Diagnostics, Mannheim, Germany).
Flórez-Moreno et al.^24^, 2013 (Colombia) Am J Orthod Dentofac Orthop (3.0) [sRANKL; OPG]	Prospective QE # Yes - Consent term; $ Research Development Committee of the University of Antioquia (No. CODI-Code 8700-1618/2009); * None.	(n = 14) (♀ = 5, ♂ = 9) 11-18 yo (N.R.)	Young group (n=14) Fixed orthodontic appliance: Roth prescription, 0.018x0.025-in brackets and 0.014-in NiTi conventional archwire. (8 weeks)	- Collection before breakfast and any dental hygiene procedure. - UWS (5mL) spit into a 50-mL sterile plastic centrifuge tube (Greiner Bio-one, Frickenhausen, Germany). - Centrifugation for 5min at 800g (IEC Centra CL2 centrifuge; Thermo Electron, Milford, Mass) and supernatants into 500-µL volumes at -75°C until processed.	- Ampli sRANKL Human ELISA and OPG ELISA (Immunodiagnostic Systems, Fountain Hills, AZ, USA); - Microplate Reader (ChroMate 4300; Awareness Technology, Palm City, FL, USA).
Bakar et al.^25^, 2017 (Malaysia) Braz J Oral Sci (N.R.) [Leptina]	Prospective QE # Yes - Consent term; $ International Islamic University Malaysia (Nos. EDW-B-14-103-0988 e RIGS16-139-0303); * N.R.	(n = 10) (♀ = 7, ♂ = 3) N.R. (16.76±1.1)	Only group (n=10) Fixed orthodontic appliance: fixed upper and lower orthodontic appliances bonded with 0.014-in NiTi wires attached to both arches. (6 weeks)	- Fast from midnight, collection at 8am before toothbrushing. - UWS (5mL) expectorated into disposable tubes every 30sec over 5min. - Centrifugation for 10min at 4000g and samples at -25°C until analyzed.	- Leptin Abnova LEP Human ELISA kit (KA3080).
Al-Khatieeb et al.^26^, 2018 (Iraq) J Contemp Dent Pract (N.R.) [ALP; LDH]	Prospective QE # N.R. - Consent term; $ None; * None.	(n = 16) (♀ = 6, ♂ = 10) 9-12 yo (N.R.)	Only group (n=16) Removable orthodontic appliance: monoblock. (28 days)	- No drinking or eating for at least 1h and sit in a comfortable position. - UWS (5mL) spit into a 10-mL sterile plastic plane test tube within 10min. - Cooling box, centrifugation for 20min at 3000RPM and supernatants in Eppendorf tubes at -20°C until analysis.	- Semi-Auto Chemistry Analyzer (BA-88A; Mindray, Nanshan, Shenzhen, China).
Aksoy et al.^27^, 2019 (Turkey) Turk J Orthod (1.1) [Cortisol]	Prospective QE # Yes - Consent term; $ Süleyman Demirel University Scientific Research Projects Unit (No. 2494-M-10); * None.	(n = 20) (♀ = 10, ♂ = 10) 11-14 yo (12.83±0.71)	G1 (n=10) | G2 (n=10) Fixed orthodontic appliance: 0.016-, 0.016×0.016-, and 0.16×0.22-in (G1) and 0.014-, 0.016-, and 0.016×0.016-in (G2) straight wires (Superelastic NiTi Memory Wire; American Orthodontics, Sheboygan, WI, USA). (8 weeks)	- No drinking (only water allowed) or eating for 1h, and sit in normal position. - Saliva collected into a polypropylene tube for 3min. - Samples at -80°C until analysis.	- Cobas Cortisol Kit (Roche Diagnostics, USA); - Cobas e601 Immunoassay System (Roche Diagnostics, Mannheim, Germany).
Maan & Patil^28^, 2019 (India) J Orthod Sci (N.R.) [IL-1β; PGE2]	Prospective QE # Yes - Consent term; $ None; * None.	(n = 10) (♀ = 5, ♂ = 5) 12-18 yo (N.R.)	Young group (n=10) Fixed orthodontic appliance: MBT prescription, preadjusted Edgewise brackets (3M Gemini brackets; 3M Unitek Corporation, Monrovia, California) with 0.022-in slots, with 0.014-in (T2), and 0.016-in (T4) NiTi archwires (Ortho Organizers Inc., USA). (1 month)	- Preparation: N.R. - UWS collected into a 45-mL sterile plastic tube. - Samples stored in 2mL Eppendorf tubes at -79°C.	- IL‐1β ELISA Kit (Krishgen Biosystems, India); - PGE2 ELISA Kit (KinesisDx, USA); - Spectrophotometric Microplate Reader (Lisa Plus, India).
Jha et al.^29^, 2020 (India) J Fam Med Prim Care (1.4) [IgA]	Prospective QE # Yes - Consent term; $ None; * None.	(n = 40) (♀ = N.R. ♂ = N.R.) 8-15 yo (N.R.)	G1 (n=20) | G2 (n=20) Fixed orthodontic appliance (G1) and removable orthodontic appliance (G2). (6 months)	- No drinking or eating for 1h, collection in the morning between 10am and 11am. - UWS spit into a pre-labeled sterile container. - 1.5mL test tubes, dry ice for transportation, and deep freezer (Samsung RZ90EERS) at-20°C.	- IgA ELISA Kit.
Grgurevic et al.^30^, 2021 (Croatia) Prog Orthod (4.8) [BMP4]	Prospective QE # Yes - Consent term; $ Scientific Center of Excellence for Reproductive and Regenerative Medicine (project “Reproductive and regenerative medicine - exploration of new platforms and potentials”) and European Union by European Regional Development Fund (No. KK.01.1.1.01.0008); * None.	(n = 18) (♂ = 18) 12-14 yo (N.R.)	G1 (n=12) | Comparator group (n=6) Fixed orthodontic appliance: MBT prescription, with 0.022-in slot and 0.012-in NiTi straight wire. (30 days)	- No eating or toothbrushing for 1h. - Saliva expectorated into clean Petri dishes. - Samples (1mL) stored in Eppendorf tubes at -80°C, pooled samples centrifugation for 10min at 12000g at 4°C, acetone precipitation, and analysis.	- RC DC Protein Assay Kit II (BioRad); - BMP4 ELISA DuoSet (#DY314, R&D Systems); - Mass Spectrometry (LTQ Orbitrap Discovery Instrument; Thermo Fischer Scientific).
Wazwaz et al.^31^, 2023 (United Kingdom) Sci Rep (4.6) [MMP8; MMP9]	Retrospective QE # Yes - Consent term; $ N.R.; * None.	(n = 16) (♀ = 6, ♂ = 10) 12-18 yo (15.2±1.6)	Only group (n=16) Fixed orthodontic appliance: MBT prescription, 0.022-in brackets (Victory-APC; 3M-Unitek) and specific sequence of archwires (0.014-in NiTi; 0.018-in NiTi; 0.017×0.025-in NiTi; and 0.019×0.025-in stainless steel). (6-18 months)	- Preparation: N.R. - UWS collected. - Centrifugation for 5min at 9200g and samples at -80°C until analysis.	- Human MMP8 e MMP9 ELISA Kit (R&D Systems); - BCA Protein Assay (Termo-Scientific); - LC-MSMS (Q-Exactive-Plus coupled to RSLCnano3000 (Termo-Scientific); - Zymography (ChemiDocTM MP Imaging System; BioRad).

N.R. = Not reported in the study; QE = Quasi-experimental study; ♀ = Female; ♂ = Male; yo = Year old; G1 = Experimental group 1; G2 = Experimental group 2; UWS = Unstimulated whole saliva; Ig A/G/M/D/E = Immunoglobulins A/G/M/D/E; Ca2+ = Calcium; Pi3+ = Phosphate; Na+ = Sodium; Cl- = Chloride; K+ = Potassium; sRANKL = Soluble receptor activator of nuclear factor Kappa B ligand; OPG = Osteoprotegerin; ALP = Alkaline phosphatase; LDH = Lactate dehydrogenase; IL-1β = Interleukin-1β; PGE2 = Prostaglandin E2; BMP4 = Bone morphogenetic protein 4; MMP 8/9 = Matrix metalloproteinases 8/9.

### RISK OF INDIVIDUAL BIAS IN THE STUDIES

**Figure 2:**
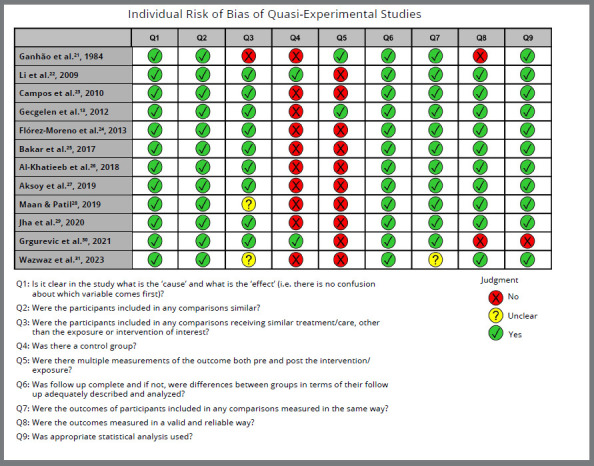
Individual risk of bias of quasi-experimental studies.

Figure 2 presents the individual assessment of each eligible study. One study^21^ included a group with a mix of participants without and with dental caries receiving treatment before starting an orthodontic movement, and it was considered different care between participants (Q3, Fig 2). One study^28^ did not report any preparation protocol before saliva collection, and another^31^ did not explicitly report the duration of orthodontic treatment; they were considered uncertain (Q3, Fig 2).

Only two studies^22, 30^ had an actual comparator group (Q4, Fig 2). Two other studies^13, 21^ had multiple pre- and post-treatment measurements (Q5, Fig 2). One study^31^ evaluated only five of all study samples (n=16) at each assessment time, and the similarity in measuring participants’ outcomes was considered uncertain (Q7, Fig 2).

One study^30^ presented significant methodological differences in measuring outcomes, compared to other studies, performing the combined centrifugation of salivary samples (Q8, Fig 2) without presenting quantitative data processed in a static test (Q9, Fig 2).

### INDIVIDUAL STUDY FINDINGS

Li et al.^22^ showed temporary changes in electrolyte concentrations in saliva in the first month of using fixed orthodontic appliances, presenting a decrease in Ca2+, Pi3+, and K+, and an increase in Na+ and Cl-.

Al-Khatieeb et al.^26^ found significant changes in ALP and LDH levels during the first month of using a removable orthodontic appliance (monoblock). Wazwaz et al.^31^ demonstrated increased MMP8 and MMP9 levels from the first hour of orthodontic tooth movement, with changes over time, and proteases and collagen-derived peptides associated with inflammatory processes.

Gecgelen et al.^13^ proposed that rapid maxillary expansion is associated with changes in patients’ anxiety and cortisol, with higher cortisol levels on the first day of orthodontic treatment and altered cortisol patterns during the first 36 days of treatment. Aksoy et al.^27^ also found higher cortisol results in the early stages of orthodontic therapy. Conversely, Bakar et al.^25^ showed a constant decrease in leptin levels, a hormone possibly related to tooth movement.

Ganhão et al.^21^ found small changes in salivary immunoglobulin concentrations, without a significant effect associated with orthodontic treatment, related to the insensitivity of the applied radial immunodiffusion test. Campos et al.^23^ also presented small changes in salivary IgA levels. They correlated these levels with oral pain intensity reported in children, outlining a possible protective role of salivary IgA in pain perception. In contrast to previous studies, Jha et al.^29^ considered removable and fixed orthodontic appliances as possible local immunogenic factors, stimulating saliva secretion and slightly higher levels of salivary IgA.

Flórez-Moreno et al.^24^ related small changes in sRANKL and OPG salivary concentrations to different orthodontic treatment stages, with fluctuating values ​​over eight weeks, significantly increasing the sRANKL/OPG ratio in the eighth week. Maan & Patil^28^ demonstrated changes in IL-1β and PGE2, with higher levels of inflammatory mediators during the initial phases of orthodontic treatment. Grgurevic et al.^30^ presented an association between orthodontic appliance placement and BMP4 identification in saliva samples, considering sample size limitations, study duration, and confounding factor assessments among participants.

Only one study^30^ did not present quantitative results. The others had their main quantitative results organized in a table (Table 3), and were categorized into five groups according to the analyzed biomarkers: (1) Electrolytes, (2) Enzymes, (3) Hormones, (4) Immunoglobulins, and (5) Mediators.

**Table 3:** Main quantitative results of eligible studies: Mean and standard deviation of biomarkers for each moment of saliva collection and analysis.

Author, year (Biomarker) [Measurement unit]	Collection time: Reference time	Results (mean ±SD) Group | Group	Group (n)
Electrolytes			
Li et al.^22^, 2009 (Ca2+) [M]	T1: baseline	1.16 ± 0.36 | 1.11 ± 0.16	G1* (n=12) | CG* (n=12)
	T2: 1mo ~	0.95 ± 0.11 | 1.07 ± 0.15	
	T3: 3mo ~	1.02 ± 0.10 | 1.01 ± 0.11	
	T4: 6mo ~	1.01 ± 0.29 | 1.04 ± 0.15	
Li et al.^22^, 2009 (Pi3+) [M]	T1: baseline	3.02 ± 0.21 | 2.99 ± 0.34	G1* (n=12) | CG* (n=12)
	T2: 1mo ~	2.11 ± 0.62 | 2.95 ± 0.15	
	T3: 3mo ~	2.74 ± 0.34 | 3.00 ± 0.30	
	T4: 6mo ~	2.82 ± 0.37 | 3.01 ± 0.10	
Li et al.^22^, 2009 (Na+) [M]	T1: baseline	9.00 ± 1.71 | 9.21 ± 1.01	G1* (n=12) | CG* (n=12)
	T2: 1mo ~	14.93 ± 1.82 | 8.86 ± 2.01	
	T3: 3mo ~	8.71 ± 1.27 | 9.72 ± 1.16	
	T4: 6mo ~	8.57 ± 1.22 | 9.88 ± 1.58	
Li et al.^22^, 2009 (Cl-) [M]	T1: baseline	21.07 ± 3.61 | 22.70 ± 3.37	G1* (n=12) | CG* (n=12)
	T2: 1mo ~	25.86 ± 4.69 | 21.30 ± 2.75	
	T3: 3mo ~	19.64 ± 2.71 | 21.30 ± 3.33	
	T4: 6mo ~	19.86 ± 1.96 | 20.30 ± 1.70	
Li et al.^22^, 2009 (K+) [M]	T1: baseline	24.51 ± 4.96 | 24.09 ± 2.93	G1* (n=12) | CG* (n=12)
	T2: 1mo ~	14.80 ± 1.49 | 22.54 ± 2.16	
	T3: 3mo ~	24.29 ± 5.22 | 23.41 ± 2.63	
	T4: 6mo ~	23.49 ± 4.64 | 23.98 ± 2.53	
Enzymes			
Al-Khatieeb et al.^26^, 2018 (ALP) [IU/L]	T1: baseline	33.94 ± 7.36	OG” (n=16)
	T2: 1h ~	42.38 ± 8.98	
	T3: 14d ~	64.00 ± 15.50	
	T4: 28d ~	104.06 ± 28.47	
Al-Khatieeb et al.^26^, 2018 (LDH) [IU/L]	T1: baseline	179.63 ± 22.55	OG” (n=16)
	T2: 1h ~	225.81 ± 33.06	
	T3: 14d ~	320.19 ± 60.64	
	T4: 28d ~	496.31 ± 98.04	
Wazwaz et al.^31^, 2023 (MMP8) [pg/mL]	T1: baseline	#9.300 ± N.R.	OG* (n=16)
	T2: 1h ~	#12.400 ± N.R.	
	T3: 1w ~	#15.500 ± N.R.	
	T4: after alignment conclusion	#12.600 ± N.R.	
Wazwaz et al.^31^, 2023 (MMP9) [pg/mL]	T1: baseline	#3.900 ± N.R.	OG* (n=16)
	T2: 1h ~	#5.000 ± N.R.	
	T3: 1w ~	#5.950 ± N.R.	
	T4: after alignment conclusion	#6.000 ± N.R.	
Hormones			
Gecgelen et al.^13^, 2012 (Cortisol) [nmol/L]	T1a: baseline (1w before; 09h15)	9.48 ± N.R.	OG* (n=40)
	T1b: baseline (1w before; 09h40)	7.85 ± N.R.	
	T1c: baseline (1w before; 10h00)	6.67 ± N.R.	
	T1d: baseline (1w before; 10h30)	4.84 ± N.R.	
	T2a: 15min before (09h15)	10.55 ± N.R.	
	T2b: 10min ~ (09h40)	13.92 ± N.R.	
	T2c: 30min ~ (10h00)	11.38 ± N.R.	
	T2d: 60min ~ (10h30)	7.26 ± N.R.	
	T3a: 1d ~ (09h15)	8.40 ± N.R.	
	T3b: 1d ~ (09h40)	8.60 ± N.R.	
	T3c: 1d ~ (10h00)	7.29 ± N.R.	
	T3d: 1d ~ (10h30)	5.51 ± N.R.	
	T4a: 4d ~ (09h15)	9.15 ± N.R.	
	T4b: 4d ~ (09h40)	10.02 ± N.R.	
	T4c: 4d ~ (10h00)	8.19 ± N.R.	
	T4d: 4d ~ (10h30)	6.47 ± N.R.	
	T5a: 7d ~ (09h15)	8.84 ± N.R.	
	T5b: 7d ~ (09h40)	8.60 ± N.R.	
	T5c: 7d ~ (10h00)	7.23 ± N.R.	
	T5d: 7d ~ (10h30)	5.65 ± N.R.	
	T6a: 15d ~ (09h15)	9.69 ± N.R.	
	T6b: 15d ~ (09h40)	9.20 ± N.R.	
	T6c: 15d ~ (10h00)	7.88 ± N.R.	
	T6d: 15d ~ (10h30)	6.27 ± N.R.	
	T7a: 25d ~ (09h15)	9.40 ± N.R.	
	T7b: 25d ~ (09h40)	9.27 ± N.R.	
	T7c: 25d ~ (10h00)	8.21 ± N.R.	
	T7d: 25d ~ (10h30)	6.82 ± N.R.	
	T8a: 36d ~ (09h15)	9.83 ± N.R.	
	T8b: 36d ~ (09h40)	9.50 ± N.R.	
	T8c: 36d ~ (10h00)	8.02 ± N.R.	
	T8d: 36d ~ (10h30)	6.07 ± N.R.	
	T9a: 3mo + (09h15)	9.28 ± N.R.	
	T9b: 3mo + (09h40)	8.04 ± N.R.	
	T9c: 3mo + (10h00)	6.41 ± N.R.	
	T9d: 3mo + (10h30)	5.19 ± N.R.	
Bakar et al.^25^, 2017 (Leptina) [pg/mL]	T1: baseline (6w before)	7016.457 ± 1344.468	OG* (n=10)
	T2: at bonding	5018.528 ± 901.327	
	T3: 6w ~	4901.923 ± 754.657	
Aksoy et al.^27^, 2019 (Cortisol) [μg/dL]	T1: baseline (just before banding)	0.39 ± 0.03 | 0.41 ± 0.04	G1* (n=10) | G2* (n=10)
	T2: 1d ~	0.51 ± 0.04 | 0.52 ± 0.06	
	T3a: 2d ~ (before brackets/arch)	0.50 ± 0.07 | 0.51 ± 0.04	
	T3b: 2d ~ (after brackets/arch)	0.57 ± 0.07 | 0.59 ± 0.03	
	T4: 7d ~	0.46 ± 0.03 | 0.44 ± 0.03	
	T5a: 4w ~ (before new arch)	0.44 ± 0.03 | 0.42 ± 0.05	
	T5b: 4w ~ (after new arch)	0.45 ± 0.05 | 0.42 ± 0.03	
	T6a: 8w ~ (before new arch)	0.38 ± 0.03 | 0.38 ± 0.03	
	T6b: 8w ~ (after new arch)	0.41 ± 0.02 | 0.40 ± 0.02	
Immunoglobulins			
Ganhão et al.^21^, 1984 (IgA) [IU/mL]			OG-*
	T1: baseline (1mo before banding)	10.3 ± 4.9	T1 (n=31)
	T2: 15min before banding	12.5 ± 3.3	T2 (n=13)
	T3: 1mo ~	9.5 ± 5.3	T3 (n=21)
	T4: 3mo +	6.5 ± 4.9	T4 (n=6)
Ganhão et al.^21^, 1984 (IgG) [IU/mL]			OG-*
	T1: baseline (1mo before banding)	0.31 ± 0.27	T1 (n=31)
	T2: 15min before banding	0.51 ± 0.47	T2 (n=13)
	T3: 1mo ~	0.67 ± 1.52	T3 (n=18)
	T4: 3mo +	0.18 ± 0.23	T4 (n=6)
Ganhão et al.^21^, 1984 (IgM) [IU/mL]			OG-*
	T1: baseline (1mo before banding)	0.43 ± 0.90	T1 (n=30)
	T2: 15min before banding	0.63 ± 1.70	T2 (n=13)
	T3: 1mo ~	0.25 ± 0.92	T3 (n=17)
	T4: 3mo +	0.87 ± 1.50	T4 (n=3)
Ganhão et al.^21^, 1984 (IgD) [IU/mL]			OG-*
	T1: baseline (1mo before banding)	Trace	T1 (n=30)
	T2: 15min before banding	Trace	T2 (n=13)
	T3: 1mo ~	Trace	T3 (n=19)
	T4: 3mo +	Trace	T4 (n=6)
Ganhão et al.^21^, 1984 (IgE) [IU/mL]			OG-*
	T1: baseline (1mo before banding)	0.07 ± 0.19	T1 (n=7)
	T2: 15min before banding	3.40 ± 8.13	T2 (n=6)
	T3: 1mo ~	0.17 ± 0.22	T3 (n=7)
	T4: 3mo +	0.30 ± 0.42	T4 (n=2)
Campos et al.^23^, 2010 (IgA) [µg/mL]	T1: baseline (1-7d)	1.211.65 ± 703.24	YG* (n=10)
	T2: at bonding (8-14d)	1.397.66 ± 940.76	
	T3: arch initial insertion (15-21d)	1.100.20 ± 703.24	
Jha et al.^29^, 2020 (IgA) [µg/mL]	T1: baseline	137.45 ± 2.5 | 139.73 ± 2.3	G1* (n=20) | G2” (n=20)
	T2: 3mo ~	164.0 ± 3.23 | 144.27 ± 5.32	
	T3: 6mo ~	166.4 ± 3.65 | 145.8 ± 6.02	
Mediators			
Flórez-Moreno et al.^24^, 2013 (sRANKL) [pg/mL]	T1: baseline	Md 2.90 (1.65-8.55)	YG* (n=14)
	T2: 24-48h ~	Md 2.70 (1.75-4.15)	
	T3: 2w ~	Md 3.10 (1.85-3.10)	
	T4: 5w ~	Md 3.10 (1.90-5.55)	
	T5: 8w ~	Md 8.40 (3.10-27.4)	
Flórez-Moreno et al.^24^, 2013 (OPG) [pg/mL]	T1: baseline	Md 96.8 (31.0-190.1)	YG* (n=14)
	T2: 24-48h ~	Md 74.3 (46.3-122.6)	
	T3: 2 w ~	Md 83.6 (41.1-203.7)	
	T4: 5 w ~	Md 77.9 (24.0-124.1)	
	T5: 8 w ~	Md 62.0 (2.8-104.4)	
Maan & Patil^28^, 2019 (IL‐1β) [pg/mL]	T1: baseline (before arch)	3.62 ± 1.03 | 4.06 ± 2.21	YG×* (n=10) ♀=5 | ♂=5
	T2: 1h ~ (after arch)	12.14 ± 2.86 | 11.26 ± 3.13	
	T3: 1mo ~ (before new arch)	6.82 ± 1.64 | 5.14 ± 0.88	
	T4: 1mo ~ (after new arch)	8.92 ± 1.44 | 7.10 ± 1.00	
Maan & Patil^28^, 2019 (PGE2) [pg/mL]	T1: baseline (before arch)	41.54 ± 9.85 | 53.46 ± 15.87	YG×* (n=10) ♀=5 | ♂=5
	T2: 1h ~ (after arch)	69.90 ± 13.17 | 134.62 ± 26.06	
	T3: 1mo ~ (before new arch)	43.18 ± 5.73 | 96.22 ± 12.70	
	T4: 1mo ~ (after new arch)	51.36 ± 5.16 | 110.42 ± 10.83	

Ca2+ = calcium; Pi3+ = phosphate; Na+ = sodium; Cl- = chloride; K+ = potassium; ALP = alkaline phosphatase; LDH = lactate dehydrogenase; MMP 8/9 = matrix metalloproteinases 8/9; Ig A/G/M/D/E = Immunoglobulins A/G/M/D/E; sRANKL = soluble receptor activator of nuclear factor Kappa B ligand; OPG = osteoprotegerin; IL‐1β = interleukin-1β; PGE2 = prostaglandin E2; M / nmol = molarity / nanomol; IU = international unit; mL / dL / L = milliliter / deciliter / liter; pg / µg / g = picogram / microgram / gram; min / h / d / w / mo = minute / hour / day / week / month; ~ = after treatment start; + = after treatment end; # = approximate result after graphic reading; Md = median and interquartile range; OG = only group; OG- = only group with loses; YG = young group; ♀ = female; ♂ = male; G1 = experimental group 1; G2 = experimental group 2; CG = comparator group; × = group with results by sex; * / ” = fixed / removable orthodontic appliance.

### CERTAINTY OF EVIDENCE (GRADE APPROACH)

The certainty of evidence analysis considered biomarker categories (electrolytes, enzymes, hormones, immunoglobulins, and mediators). The outcomes presented a very low certainty level. Table 4 details the individual assessment of each outcome.

**Table 4:** Summary of findings by the Grading of Recommendations Assessment, Development, and Evaluation (GRADE) for the systematic review outcomes.

GRADE assessment		
Number of studies (participants)	Summary of findings	Certainty
Effects of orthodontic treatment on salivary electrolytes		
1 (24 participants)	The evidence showed little effect of the treatment on increasing Ca, Pi and K and reducing Na and Cl, only after the first month. The control group remained stable throughout the study period.	⨁ Very low^a,b,c^
Effects of orthodontic treatment on salivary enzymes		
2 (32 participants)	The evidence showed moderate effect of the treatment on increasing ALP and LDH during the first month, and increasing MMP8 and MMP9 during all treatment period.	⨁ Very low^a,b,d^
Effects of orthodontic treatment on salivary hormones		
3 (70 participants)	The evidence showed little effect of rapid maxillary expansion on increasing cortisol during all expansion period. However, the evidence also showed no effect of treatment with fixed orthodontic appliances on cortisol levels. Regarding leptin levels, evidence showed little reduction six weeks after treatment.	⨁ Very low^a,b^
Effects of orthodontic treatment on salivary immunoglobulins		
3 (81 participants)	The evidence showed little to no effect of orthodontic treatment on salivary immunoglobulin concentrations.	⨁ Very low^a,b,d^
Effects of orthodontic treatment on mediators		
2 (24 participants)	The evidence showed nonlinear trends in the levels of the biomarkers through time.	⨁ Very low^a,b,d^

a = Non-randomized study - downgraded by two levels. b = Imprecision - Very few participants (<100) - downgraded by two levels. c = Inconsistency was not assessed because there was only one study included. d = One or more studies presented serious sources of risk of bias - downgraded by one level. **GRADE Working Group grades of evidence**. High certainty = Very confident that the true effect is close to the estimated effect. Moderate certainty = Moderately confident in the estimated effect - The true effect is likely close to the estimated effect, but it may be substantially different. Low certainty = Limited confidence in the estimated effect - The true effect may be substantially different from the estimated effect. Very low certainty = Very little confidence in the estimated effect - The true effect may be substantially different from the estimated effect.

## DISCUSSION

Relevant findings were obtained after systematically analyzing the literature on changes in endogenous salivary biomarkers of pain, anxiety, stress, and inflammation related to tooth movement during orthodontic treatment in children and adolescents. The results are promising for using different biomarkers to monitor and predict orthodontic stages and adverse effects. However, the literature on salivary biomarkers and orthodontic movement is still scarce compared to gingival crevicular fluid biomarkers.

The first eligible study for this review was published in the 1980s, and the others were developed in the last two decades. This content is still recent, coinciding with findings from other reviews on salivary biomarkers in orthodontics.^2^ ^,^ ^32^ ^,^ ^33^ That may be associated with the overlap of publications on gingival crevicular fluid biomarkers perpetuated for years with extensive and developing literature.^34^

Each study evaluated different salivary biomarkers, with literature reviews on gingival crevicular fluid biomarkers also reporting heterogeneity.^35^ ^,^ ^36^ Thus, the biomarkers were organized according to their nature and biological function, with an evident and coherent presentation in mind. The literature categorizes biomarkers according to their nature,^36^ ^,^ ^37^ primary biological function,^38^ ^,^ ^39^ orthodontic treatment phases,^34^ and criteria mixtures.^35^ ^,^ ^36^ ^,^ ^40^

The temporary changes in electrolytes were correlated with the increased mechanosensation from wearing fixed orthodontic appliances, an adaptation of patients’ daily routine during the first month, and changes in saliva flow rate.^22^ The significant decrease in Ca+, Pi3+, and K+ salivary concentration in the first month^22^ may present a greater risk of dental caries, affecting the demineralization-remineralization process. Recent systematic reviews suggest associations between oxidative stress^41^ and unbalanced salivary components^42^ with dental caries. Therefore, oral environment adaptation before orthodontic treatment and good oral health guidelines must be carried out thoroughly to avoid potential risks. Removable aligners, instead of fixed appliances, can positively influence the reduction of plaque accumulation.^43^

The increased salivary MMP^31^ related to inflammatory processes from orthodontic movement can also increase the risk of dental caries. A literature review recently described the relationship of MMP with dentin caries due to dentin collagen matrix degradation.^44^ Two correlations were proposed regarding the more significant activity of ALP and LDH: the mechanical forces of tooth movement generated by the monoblock appliance or the rapid growth phase of children in late childhood and early puberty. ^26^ Furthermore, the significant LDH increase in the first hour^26^ may relate to the latency phase of orthodontic movement, marked by the role of inflammatory mediators of apoptosis.^34^ Meanwhile, the more discrete and progressive ALP increase^26^ may represent osteoblasts’ cellular activation and differentiation.^34^

Salivary cortisol increased on the first day of treatment,^13, 27^ which continued to change for 36 days.^13^ Therefore, orthodontic treatment may relate to stress and anxiety, but other biopsychosocial factors should probably be evaluated together, for example, the academic stress of students.^45^ The significant decrease in leptin^25^ may be due to its anti-inflammatory capacity inhibited by the inflammatory mediators of tooth movement. Leptin level changes are also associated with overweight and periodontal disease.^46^ Individualized approaches from the expanded context may be designed with multidisciplinary and multi-professional articulations for each case.

Among salivary immunoglobulin findings, IgA presented controversial results between studies,^21^ ^,^ ^23^ ^,^ ^29^ remaining a biomarker with an uncertain association regarding orthodontic movement. The literature describes salivary IgA as a potential biomarker for pain.^47^ Thus, salivary IgA changes may interact with oral pain perception in orthodontic patients. Pain and discomfort are expected impacts during orthodontic treatment, with a tendency to adapt over time.^48^ Therefore, monitoring salivary IgA and assessing oral health-related quality of life may be a promising research field for improving orthodontic treatment.

The significant increase in IL-1β and PGE2 in the early stages^28^ reflects the initial inflammatory action of orthodontic movement. Higher levels of salivary IL-1β may be associated with higher pain intensity.^49^ The fluctuations in sRANKL and OPG values ​​during the first eight weeks^24^ and the presence of salivary BMP4^30^ may be related to the biological phenomena of bone reabsorption and formation. The higher sRANKL/OPG ratio in the eighth week^24^ represents the protagonism of bone remodeling after the previous phases.^34^ The literature associates higher RANKL/OPG ratios with higher orthodontic movement speed.^49^ Hence, these biomarkers potentially monitor phases and predict treatment aspects.

This review has some limitations. First, the methodological heterogeneity of selected studies did not allow a meta-analysis. Despite the reasonable number of selected studies (n=12), only five analyzed the same biomarker: IgA in three studies^21^ ^,^ ^23^ ^,^ ^29^ and cortisol in two^13^ ^,^ ^27^ . Even so, they still had different saliva collection times and analysis methods. Second, most studies lacked an actual comparator group and multiple measurements, hampering more robust data analysis and accurate considerations on changes associated with orthodontic tooth movement. Third, most studies often diminished or neglected clinical contexts and possible confounders, reinforcing the need for caution in interpreting results.

Although there was a high heterogeneity among the selected studies, the systematic approach of this review allowed a comprehensive, extensive, cautious, and well-organized data presentation on the topic. That provides a proper acknowledgment of findings to promote evidence-based clinical guidance and orientation, also serving as a literature gap identifier for future studies. It is worth noting that most studies presented a low risk of bias and were well-conducted within their circumstances, such as resources, ethical criteria, sampling, and outcomes.

The salivary levels of stress, pain, anxiety, and inflammation biomarkers are relevant in understanding patients’ physiological responses to orthodontic interventions. These findings may aid clinical routine and allow orthodontists to monitor these parameters with salivary diagnostic tests, which may provide data on their patient’s health status and assist in individualizing treatment plans. Thus, knowing these variations may improve decision-making based on patients’ well-being throughout the treatment.

## CONCLUSION

Based on a very low certainty level, orthodontic tooth movement had little to no effect on endogenous salivary biomarkers. Further studies with standardized methods should be performed to improve the understanding of possible confounders and effects of this association.
